# Finite mixtures of functional graphical models: Uncovering heterogeneous dependencies in high-dimensional data

**DOI:** 10.1371/journal.pone.0316458

**Published:** 2025-01-02

**Authors:** Qihai Liu, Kevin H. Lee, Hyun Bin Kang

**Affiliations:** Department of Statistics, Western Michigan University, Kalamazoo, MI, United States of America; Indian Statistical Institute, INDIA

## Abstract

Graphical models have been widely used to explicitly capture the statistical relationships among the variables of interest in the form of a graph. The central question in these models is to infer significant conditional dependencies or independencies from high-dimensional data. In the current literature, it is common to assume that the high-dimensional data come from a homogeneous source and follow a parametric graphical model. However, in real-world context the observed data often come from different sources and may have heterogeneous dependencies across the whole population. In addition, for time-dependent data, many work has been done to estimate discrete correlation structures at each time point but less work has been done to estimate global correlation structures over all time points. In this work, we propose finite mixtures of functional graphical models (MFGM), which detect the heterogeneous subgroups of the population and estimate single graph for each subgroup by considering the correlation structures. We further design an estimation method for MFGM using an iterative Expectation-Maximization (EM) algorithm and functional graphical lasso (fglasso). Numerically, we demonstrate the performance of our method in simulation studies and apply our method to high-dimensional electroencephalogram (EEG) dataset taken from an alcoholism study.

## 1 Introduction

Functional data analysis (FDA) [1–4] is a rapidly developing area of statistics for data that can be naturally viewed as a smooth curve or function. Unlike traditional methods where the basic statistical unit is a vector of measurements, FDA treats entire functions or curves as the primary objects of analysis [5, 6]. With the development of data collection technologies that use powerful monitoring devices and computational tools, many scientific fields are now generating increasingly complex, high-dimensional datasets [7]. Analyzing these datasets, which can be viewed as functions, requires characterizing the relationships among numerous variables to gain insight into underlying phenomena [8].

Graphical models have been widely used to explicitly capture the statistical relationships between the variables of interest in the form of a graph. Recent progress in graphical modeling has focused on methods for modeling complex dependencies among binary variables through Ising models [9–11] and among continuous variables through Gaussian graphical models [12–16]. However, there has been less attention paid to functional variables, and most existing work concentrates on estimating discrete correlation structures at individual time points rather than global dependencies across all time points.

To address this gap, functional graphical models have been introduced to model the conditional dependence structure among random functions, such as measurements over time or frequency in data like electroencephalogram (EEG) or functional magnetic resonance imaging (fMRI). These models have been estimated using various approaches, including parametric approaches based on Gaussian assumption [17], nonparametric approaches based on the additive conditional independence or additive principal scores [18, 19], and Bayesian approaches [20]. Recent extensions are primarily based on Gaussian assumption. A doubly functional graphical model has been developed to deal with the case where functional data is sparsely observed [21]. A functional copula Gaussian graphical model was proposed to deal with marginal violation of the Gaussian assumption [22]. A conditional functional graphical models was also introduced for the graph structure that is conditioned on and thus varies with the external variables [23]. All of these approaches assume that the multivariate functional data come from a homogeneous source.

In contrast, many real-world scenarios involve data from heterogeneous sources, where dependencies may vary across different groups or subpopulations. Although it is common in graphical model literature to assume homogeneity, there has been growing interest in incorporating heterogeneity. For the continuous variables, mixtures of Gaussian graphical models and its variants have been proposed [24, 25], while for the binary variables, mixtures of Ising graphical models have been developed [26, 27]. Similarly, mixtures of ordinal graphical models have been introduced for ordinal data [28].

In this paper, we propose finite mixtures of functional graphical models (MFGM) to capture the heterogeneous conditional dependence relationships in multivariate functional data. Our method simultaneously identifies latent subgroups of the studied population and estimates separate functional graphical models for each subgroup, allowing for different dependency structures across the groups. To estimate the model, we adopt a penalized likelihood approach for sparse estimation, which involves regularizing the likelihood function with a non-smooth penalty. This creates a challenging optimization problem, especially due to the functional nature of the data. To tackle this, we extend the framework for the functional graphical model [17], assuming that the observed functional data are realizations from a Gaussian process, and propose an effective EM algorithm that incorporates the functional graphical lasso (fglasso) method.

## 2 Method

### 2.1 Mixtures of functional graphical models

Our proposed mixture of functional graphical models (MFGM) are generalization of mixture of graphical models from finite vector-valued context to infinite functional context. Suppose the functional variables *g*_1_(*t*), …, *g*_*p*_(*t*) jointly follow a *p*-dimensional multivariate Gaussian process with vertex set *V* = 1, …, *p* and edge set *E*. Let *K* be the number of mixtures and let *G*_*k*_ = (*V*, *E*_*k*_) represents the functional graphical model in the *k*th subpopulation. Now our mixture of functional graphical model can be represented as

$$G\left(\text{X}\right)=\pi_{1}G_{1}\left(\text{X}\right)+\pi_{2}G_{2}\left(\text{X}\right)+\cdots +\pi_{K}G_{K}\left(\text{X}\right),$$

where $\sum_{k=1}^{K}\pi_{k}=1$. Therefore, the goal of MFGM is to estimate ***π*** = (*π*_1_ … *π*_*K*_) and recover {*E*_1_, …, *E*_*K*_} and then infer membership label of each individual via maximizing the penalized log-likelihood of the observed functional data.

Suppose we observe ***g***_*i*_ = (*g*_*i*1_, …, *g*_*ip*_)^⊤^, *i* = 1, …, *N* and for each *i*, *g*_*ij*_(*t*), t∈T is a realization from a Gaussian process. The Karhunen-Loève expansion allows us to represent each functional variable with

$$g_{ij}\left(t\right)=\sum_{l=1}^{\infty }a_{ijl}\phi_{jl}\left(t\right),$$

for *i* = 1, …, *N* and *j* = 1, …, *p*.

We propose to approximate *g*_*ij*_(*t*) by truncating the number of bases, denoted as *M*, which increases asymptotically as *N* → ∞. the *M*-truncated version of Karhunen-Loève expansion would be

$$g_{ij}\left(t\right)\approx \sum_{l=1}^{M}a_{ijl}\phi_{jl}\left(t\right),$$

for *i* = 1, …, *N* and *j* = 1, …, *p*. Here we assume that the truncated multivariate random vector follows mixture of multivariate Gaussian distribution and

$${\text{a}}_{i}^{M}={\left({\left({\text{a}}_{i1}^{M}\right)}^{⊤},\ldots ,{\left({\text{a}}_{ip}^{M}\right)}^{⊤}\right)}^{⊤}\in R^{Mp}∼\sum_{k=1}^{K}\pi_{k}N\left(\mu_{k},\Theta_{k}\right),$$

represents the first *M* principal component scores for the *i*th set of functions for *i* = 1, …, *N*, where ${\text{a}}_{ij}^{M}=(a_{ij1},\ldots ,a_{ijM}{)}^{⊤}$. Here **Θ**_*k*_ represents the precision matrix.

Now the log-likelihood function for the observed functioanl data is given by

$$\ell =\sum_{i=1}^{N}\text{log}\sum_{k=1}^{K}\pi_{k}N\left({\text{a}}_{i}^{M}|\mu_{k},\Theta_{k}\right),$$

where

$$N\left({\text{a}}_{i}^{M}|\mu_{k},\Theta_{k}\right)={\left(2\pi \right)}^{-\frac{Mp}{2}}{\left|\Theta_{k}\right|}^{\frac{1}{2}}\;\text{exp}\;\{-\frac{1}{2}{\left({\text{a}}_{i}^{M}-\mu_{k}\right)}^{⊤}\Theta_{k}\left({\text{a}}_{i}^{M}-\mu_{k}\right)\}.$$

Given the log-likelihood, we then maximize the penalized log-likelihood to estimate *π*_*k*_, ***μ***_*k*_, and **Θ**_*k*_ for *k* = 1, …, *K* as follows:

$$\underset{\left\{\left(\pi_{k},\mu_{k},\Theta_{k}\right);k=1,\ldots ,K\right\}}{\text{max}}\;\sum_{i=1}^{N}\text{log}\sum_{k=1}^{K}\pi_{k}N\left({\text{a}}_{i}^{M}|\Theta_{k}\right)-\sum_{k=1}^{K}\lambda_{k}\sum_{j\neq l}{\left\|\Theta_{kjl}\right\|}_{F}$$

where ${\left\|\cdot \right\|}_{F}$ denotes Frobenius norm. Here, **Θ**_*kjl*_’s are *M* × *M* matrices for *j* = 1, …, *p* and *l* = 1, …, *p*.

### 2.2 Computation

The EM algorithm provides a powerful tool to deal with latent variables in mixture models. Following the spirit of the EM algorithm, we view the functional data to be incomplete, and treat the latent variables as “missing data”. Moreover, unlike traditional approaches, the sparse estimation imposes the non-smooth penalty function to regularize the likelihood function, which leads to solving a challenging non-convex and non-smooth optimization problem.

We introduce the latent random variables ***τ***_*i*_ = (*τ*_*i*1_, …, *τ*_*iK*_), *i* = 1, …, *N*, satisfying that

$$\begin{array}{c} \tau_{ik}=\{\begin{array}{cc} 1 & \;\text{if}\;g_{i}\left(t\right)\;\text{belongs}\;\text{to}\;\text{the}\;k\text{th}\;\text{group},\\ 0 & \;\text{otherwise}. \end{array} \end{array}$$ (1)

Now given the complete data, the complete log-likelihood would be

$$\ell_{\text{comp}}=\sum_{i=1}^{N}\sum_{k=1}^{K}\tau_{ik}\;\text{log}\;\pi_{k}+\tau_{ik}\;\text{log}\;N\left({\text{a}}_{i}^{M}|\mu_{k},\Theta_{k}\right),$$

and the complete *ℓ*_1_-penalized log-likelihood function becomes:

$$\begin{array}{c} L^{\text{comp}}=\ell_{\text{comp}}-\sum_{k=1}^{K}\lambda_{k}\sum_{j\neq l}{\left\|\Theta_{kjl}\right\|}_{F} \end{array}$$ (2)

**E-step**: Let $\pi_{k}^{\left(l\right)}$, $\mu_{k}^{\left(l\right)}$, and **Θ**_*k*_^(*l*)^ be the estimate of *π*_*k*_, ***μ***_*k*_, and **Θ**_*k*_ for *k* at the *l*th iteration. In the E-step of the (*l* + 1)th iteration, we compute the conditional expectation of *τ*_*ik*_ given current estimates $\pi_{k}^{\left(l\right)}$, $\mu_{k}^{\left(l\right)}$, and $\Theta_{k}^{\left(l\right)}$ for *k* = 1, …, *K*. By using Bayes’ rule, we have

$$\gamma_{ik}^{\left(l+1\right)}=\frac{\pi_{k}^{\left(l\right)}N\left({\text{a}}_{i}^{M}|\mu_{k}^{\left(l\right)},\Theta_{k}^{\left(l\right)}\right)}{\sum_{k=1}^{K}\pi_{k}^{\left(l\right)}N\left({\text{a}}_{i}^{M}|\mu_{k}^{\left(l\right)},\Theta_{k}^{\left(l\right)}\right)}.$$

**M-step**: In the M-step of the (*l* + 1)th iteration, we obtain the estimates of parameters from maximizing

$$\sum_{k=1}^{K}[\sum_{i=1}^{N}\gamma_{ik}^{\left(l+1\right)}(\text{log}\;\pi_{k}+\text{log}\;N\left({\text{a}}_{i}^{M}\right|\mu_{k},\Theta_{k})-\lambda_{k}\sum_{j\neq l}{\left\|\Theta_{kjl}\right\|}_{F}].$$

subject to the constraint that $\sum_{k=1}^{K}\pi_{k}=1$. It is equivalent to maximizing

$$\sum_{i=1}^{N}\sum_{k=1}^{K}\gamma_{ik}^{\left(l+1\right)}\;\text{log}\;\pi_{k}$$

subject to $\sum_{k=1}^{K}\pi_{k}=1$, and

$$\sum_{k=1}^{K}[\sum_{i=1}^{N}\gamma_{ik}^{\left(l+1\right)}\;\text{log}\;N\left({\text{a}}_{i}^{M}|\mu_{k},\Theta_{k}\right)-\lambda_{k}\sum_{j\neq l}{\left\|\Theta_{kjl}\right\|}_{F}].$$

for *k* = 1, … *K*.

Now by solving the two above subproblems respectively for $\pi_{k}^{\left(l+1\right)}$ and $\mu_{k}^{\left(l+1\right)}$, we can find the following closed-form solutions. We update $\pi_{k}^{\left(l+1\right)}$ by

$$\pi_{k}^{\left(l+1\right)}=\frac{1}{N}\sum_{i=1}^{N}\gamma_{ik}^{\left(l+1\right)},$$

and update $\mu_{k}^{\left(l+1\right)}$ by

$$\mu_{k}^{\left(l+1\right)}=\frac{\sum_{i=1}^{N}\gamma_{ik}^{\left(l+1\right)}{\text{a}}_{i}^{M}}{\sum_{i=1}^{N}\gamma_{ik}^{\left(l+1\right)}}.$$

Next, we update $\Theta_{k}^{\left(l+1\right)}$ by solving the below optimization problem with the state-of-art optimization algorithm fglasso [17].

$$\Theta_{k}^{\left(l+1\right)}=\text{arg}\;\underset{\Theta_{k}^{\left(l\right)}}{\text{max}}\;\{\text{log}|\Theta_{k}^{\left(l\right)}|-\text{tr}\left({\text{S}}_{k}^{\left(l+1\right)}\Theta_{k}^{\left(l\right)}\right)-\lambda_{k}\sum_{j\neq l}{\left\|\Theta_{kjl}^{\left(l\right)}\right\|}_{F}\},$$

where

$$S_{k}^{\left(l+1\right)}=\frac{\sum_{i=1}^{N}\gamma_{ik}^{\left(l+1\right)}\left({\text{a}}_{i}^{M}-\mu_{k}^{\left(l+1\right)}\right){\left({\text{a}}_{i}^{M}-\mu_{k}^{\left(l+1\right)}\right)}^{⊤}}{\sum_{i=1}^{N}\gamma_{ik}^{\left(l+1\right)}}.$$

Another way to update $\Theta_{k}\in R^{Mp\times Mp}$ is by employing the alternating direction method of multipliers (ADMM) algorithm [29] with the separability assumption on the precision matrix [30]. ADMM algorithm is useful in estimating a sparse precision matrix [31], and partial separability assumption allows the covariance matrices across different dimensions of Karhunen-Loéve expansion so instead of estimating *Mp* × *Mp*, it becomes the estimation for *Mp*^2^. The plot (c) in Fig 1 of [30] shows an example of such a precision matrix.

To further clarify the distinction between the fglasso method and the partial separability assumption, let {*θ*_*ijuvk*_: *i*, *j* = 1, …, *p*; *u*, *v* = 1, …, *M*} be the elements of $\Theta_{k}\in R^{Mp\times Mp}$. Under the partial separability assumption, we impose that all *θ*_*ijuvk*_ = 0 whenever *u* ≠ *v*. In contrast, the fglasso method applies a group lasso penalty, which encourages the parameters *θ*_*ijuvk*_ to exhibit a block structure. Specifically, all *θ*_*ijuvk*_ with *i* ≠ *j* are either simultaneously zero or nonzero.

We alternate between the E-step and the M-step until the estimates of parameters converge. Our proposed EM algorithm satisfies an ascent property as the classical EM algorithm and the proof follows [25]. Here, the ascent property means the likelihood value will not decrease after each step of EM. However, the ascent property does not imply that the EM updates will necessarily converge to the MLE and our proposed EM algorithm may converge to a local maximum of the observed data likelihood function, depending on the initial values. The EM algorithm is sensitive to the initial values of the parameters, so care must be taken in the first step. In this work, the Mclust function, acquired from the R package mclust, and the split_comp function, acquired from the R package gmgm, are applied to the multivariate principal component score vectors to provide good initials for the EM algorithm.

Now we discuss the tuning parameter selection of our algorithm via a cross-validation (CV) approach. The *J*-fold CV score for *K*-mixture case is represented with:

$$\begin{array}{c} CV\left(\lambda_{1},\ldots ,\lambda_{K}\right)=\sum_{j=1}^{J}\sum_{k=1}^{K}N_{j}(\text{log}\;{\hat{\pi }}_{k,-j}-\text{log}\left|{\hat{\Theta }}_{k,-j}^{\lambda_{k}}\right|+\text{tr}\left({\hat{\Theta }}_{k,-j}^{\lambda_{k}}\Sigma_{k,j}\right)), \end{array}$$ (3)

where *N*_*j*_ is the sample size of test data in *j*th CV, ${\hat{\pi }}_{k,-j}$ is the estimated *k*th group proportion by using training data in *j*th CV, ${\hat{\Theta }}_{k,-j}^{\lambda_{k}}$ is the estimated precision matrix of *k*th group by using training data with the tuning parameter *λ*_*k*_ in *j*th CV, and **Σ**_*k*,*j*_ is the test data sample covariance matrix in *j*th CV. This cross-validation score approximates negative log-likelihood of the data. Therefore, a lower cross-validation score indicates better estimation. We built on the cross-validation score for penalized likelihood estimation in Gaussian graphical models [32] and extended it to accommodate mixtures of distributions. As the regular grid search process requires too much computing time for finding the optimal tuning parameters, the more efficient random search process is performed to find the optimal tuning parameter vector (λ_1_, …, λ_*K*_)^⊤^ that results in smallest value of CV. Then, the optimal tuning parameter vector is used for MFGM to estimate parameters.

## 3 Results and discussion

### 3.1 Simulation study

We conduct a series of simulations to compare our MFGM algorithm with fglasso algorithm and ADMM algorithm under the partial separability assumption. For simplicity, we refer to these methods as MFGM-fglasso and MFGM-ADMM, respectively. The MFGM-ADMM implementation is based on the R package fgm. Additionally, we compare these two methods with the mixggm algorithm [33], which ignores the functional structure. We take the average of observations across the time interval for each node, making a functional object into a single value, and implements mixture of Gaussian graphical models in a multivariate vector context. The implementation of mixggm algorithm is based on the R package mixggm.

#### 3.1.1 Simulation settings

In each setting, the multivariate Gaussian functional variables are generated via *g*_*ij*_ = **s**(*t*)^⊤^ ***δ***_*ij*_ for *i* = 1, …, *N* and *j* = 1, …, *p*, where **s**(*t*) is a five-dimensional Fourier basis function, and $\delta_{ij}\in R^{5}$ is a mean zero Gaussian random vector. Hence, $\delta_{i}={\left(\delta_{i1}^{⊤},\ldots ,\delta_{ip}^{⊤}\right)}^{⊤}\in R^{5p}$ follows a multivariate Gaussian distribution with covariance **Σ** = **Θ**^−1^ [17]. Different block sparsity patterns in the precision matrix **Θ** correspond to different conditional dependence structures. We consider five general structures as follows:

- Model 1 (Independent): An identity precision matrix of dimension 5*p* × 5*p* is generated. Hence, all of the *p* nodes are disconnected. This is called Independent model.
- Model 2 (AR1): A block banded matrix **Θ** is generated with **Θ**_*jj*_ = **I**_5_ for *j* = 1, …, *p*, **Θ**_*j*,*j*+1_ = **Θ**_*j*+1,*j*_ = 0.5**I**_5_ for *j* = 1, …, *p* − 1, and 0 at all other locations. Hence, only the adjacent two nodes are connected. This is called Autoregressive One (AR1) model.
- Model 3 (AR2-weak): A block banded matrix **Θ** with **Θ**_*jj*_ = **I**_5_ for *j* = 1, …, *p*, **Θ**_*j*,*j*+1_ = **Θ**_*j*+1,*j*_ = 0.4**I**_5_ for *j* = 1, …, *p* − 1, **Θ**_*j*,*j*+2_ = **Θ**_*j*+2,*j*_ = 0.2**I**_5_ for *j* = 1, …, *p* − 2, and 0 at all other locations. Hence, the consecutively adjacent three nodes are pair-wise connected. This is called Autoregressive Two (AR2) model with weak connection.
- Model 4 (AR2-strong): Similar to Model 3, a block banded matrix **Θ** is generated with **Θ**_*jj*_ = **I**_5_ for *j* = 1, …, *p*, **Θ**_*j*,*j*+1_ = **Θ**_*j*+1,*j*_ = 0.6**I**_5_ for *j* = 1, …, *p* − 1, **Θ**_*j*,*j*+2_ = **Θ**_*j*+2, *j*_ = 0.35**I**_5_ for *j* = 1, …, *p* − 2, and 0 at all other locations. Hence, the consecutively adjacent three nodes are pair-wise connected. This is called Autoregressive Two (AR2) model with strong connection.
- Model 5 (Random): A block banded matrix **Θ** is generated with random sparse connection structure: **Θ**_*jj*_ = **I**_5_ and **Θ**_*j*,*l*_ = **Θ**_*l*,*j*_ = 0.25*B*_*j*,*k*_**I**_5_ for *j*, *l* = 1, …, *p*, and *j* ≠ *l*, where *B*_*j*,*l*_ is a Bernoulli random variable which takes the value 1 with probability 0.05. The precision matrix **Θ** is generated to ensure it satisfies the positive-definite condition. This is called Random model.

**Fig 1:**
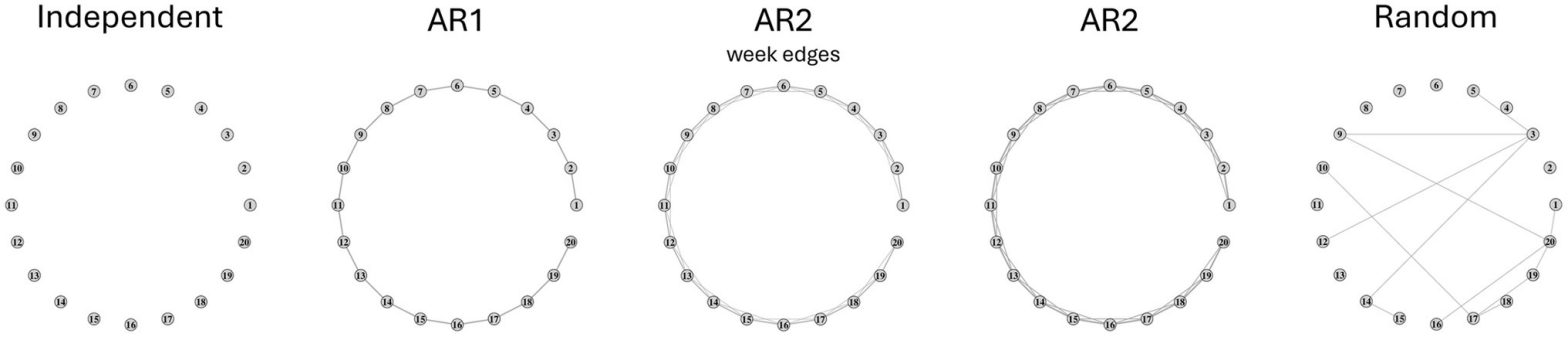
Conditional dependencies in simulated functional graphical models. Thickness of the edge denotes strength of connection.

The five simulation models are depicted in Fig 1. In all settings, we consider dimension parameter *p* = 20, and generate observations of ***δ***_*i*_ from the associated multivariate Gaussian distribution, and the observed values *h*_*ijl*_ are sampled using

$$h_{ijl}=g_{ij}\left(t_{l}\right)+e_{ijl},\;e_{ijl}∼N\left(0,0.5^{2}\right),$$

for *i* = 1, …, *N*, *j* = 1, …, *p*, and *l* = 1, …, *T* where each function is observed at *T* = 100 equally spaced time points between 0 and 1.

*Two-cluster mixture models*. We consider the following three different cases of two-cluster mixture models with $\pi =(\frac{1}{2},\frac{1}{2})$.

1. Mixture of Independent and AR2-strong (Model(1,4))
2. Mixture of AR1 and AR2-weak (Model (2,3))
3. Mixture of AR2-strong and Random (Model (4,5))

We generate *N* = 100 functional observations of ***h***_*i*_ for each mixture. We expect that it is less challenging to do clustering and to estimate connection structures in Model (1,4) as there is an obvious distinction between the Identity precision matrix and AR2 precision matrix with strong connections. Model (2,3) will be more difficult since the AR1 precision matrix and AR2 precision matrix with weak connections are more similar to each other. We go further to set the design of Model (4,5) to explore whether our method can perform good analysis in the mixture model in which a subgroup with random connection structure is involved.

*Three-cluster mixture models*. To explore even more complex scenarios, we consider the following two different cases of three-cluster mixture models with $\pi =(\frac{1}{3},\frac{1}{3},\frac{1}{3}$).

1. Mixture of Independent, AR1 and AR2-strong (Model (1,2,4))
2. Mixture of AR1, AR2-strong, and Random (Model (2,4,5))

We generate *N* = 50 functional observations of ***h***_*i*_ for each mixture. In Model (1,2,4), the three basic graphical structures, Independent, AR1, and AR2, are involved; and in Model (2,4,5), the subgroup with random graphical structure is considered for the mixture with two other heterogeneous subgroups. Here, we expect that the three-cluster mixture models are more challenging than the two-cluster mixture models to analyze.

#### 3.1.2 Simulation results

To apply our proposed MFGM algorithm to the analysis of simulated mixture data, first, the total functional observations are fitted by using an *L*-dimensional cubic B-spline basis. The Generalized Cross Validation (GCV) method is used to choose the optimal dimension parameter *L*. Then the smoothed functions are decomposed by *M*-truncated Karhunen-Loève expansion, and the optimal harmonic number *M* is determined by eight-fold CV. It turns out that *M* = 5, which aligns with our design. Further analysis reveals that five principal components already explain over 99% of the total variation in the signal trajectories for each node. The multivariate Karhunen-Loève expansion basis coefficient (principal component score) vectors $a_{i}^{M}$ with *M* = 5 are thus acquired for further mixture analysis assuming Gaussianity.

In the iterative EM process to analyze the mixture of blocked Gaussian multivariate graphical models, our proposed method utilizing the fglasso algorithm (MFGM-fglasso) is compared with the ADMM algorithm under the partial separability assumption (MFGM-ADMM), to solve the maximization problem of log-likelihood with penalty for estimating the conditional dependence structures in each cluster. Our MFGM algorithms are also compared with the mixggm algorithm to confirm the advantage of considering inherent functional nature of the data. To provide good initials for the EM iterations, the Mclust function, acquired from the R package mclust, and the split_comp function, acquired from the R package gmgm, are applied to the multivariate principal component score vectors, for two-cluster and three-cluster mixture models analysis, respectively. We tried tuning parameter values for λ_*k*_ in the range from 0.8 to 2.5, with increments of 0.1, and determined the optimal value for each group *k* by minimizing the cross-validation score in Eq (3). The optimal values were mostly from 0.9 to 1.5.

The estimation of the edge structures in each cluster are checked with following metrics; Accuracy (Accu), True Positive Rate (TPR), and False Positive Rate (FPR). We run each simulation 100 times for two-cluster mixture models analysis and 50 times for three-cluster mixture models analysis, and the means of all metrics for the three methods are obtained for comparison.

*Two-cluster mixture models analysis*. Table 1 shows the performance of estimates of the conditional dependence structures in each subgroup in the designed two-cluster mixture models. In the analysis of Model (1,4), all of the three methods do a good job to estimate the edge structure in subgroup 1. The MFGM-fglasso and mixggm outperform MFGM-ADMM in estimating the edge structure in subgroup 2. In analyzing the challenging mixture model, Model (2,3), the three methods show similar decent performances, which are a little worse than that in analyzing Model (1,4). However, in analyzing Model (4,5), MFGM-fglasso and mixggm algorithm do a decent job in estimating the conditional dependencies in both of the two subgroups, but the MFGM-ADMM suffered in estimating the conditional dependencies in subgroup 1.

**Table 1:** Comparison of edge estimations by MFGM-fglasso, MFGM-ADMM, and mixggm in two-cluster mixture simulations.

		Subgroup 1			Subgroup 2		
		Accu	TPR	FPR	Accu	TPR	FPR
Model (1,4)	MFGM-fglasso	0.9911	0.9924	0.0085	0.8450	0.7637	0.1326
	MFGM-ADMM	1.0000	1.0000	0.0000	0.7854	0.0868	0.0000
	mixggm	0.9484	1.0000	0.0543	0.8692	0.8889	0.1369
Model (2,3)	MFGM-fglasso	0.8462	0.3838	0.0117	0.8458	0.9959	0.1797
	MFGM-ADMM	0.8150	0.2130	0.0000	0.8943	0.2707	0.0000
	mixggm	0.8694	0.6243	0.0553	0.8868	0.9166	0.1183
Model (4,5)	MFGM-fglasso	0.8378	0.7483	0.1347	0.9503	0.5768	0.0035
	MFGM-ADMM	0.7858	0.0887	0.0000	0.9400	0.4545	0.0000
	mixggm	0.8614	0.8455	0.1337	0.9064	0.6614	0.0633

*Three-cluster mixture models analysis*. Table 2 compares the three algorithms in estimating the conditional dependence structures in each subgroup in the designed three-cluster mixture models. It shows that the three algorithms do better for Model (1,2,4) than for Model (2,4,5) in estimating the graphical structures in the first two subgroups. However, they do worse in estimating the graphical structure for the third subgroup. Moreover, it is revealed that MFGM-fglasso does the best to estimate the heterogeneous networks in terms of Accuracy for most of the three subgroups in both mixture models. It is worth to note that the mixggm algorithm performed similarly to MFGM-fglasso.

**Table 2:** Comparison of edge estimations by MFGM-fglasso, MFGM-ADMM, and mixggm in three-cluster mixture simulations.

		Model (1,2,4)			Model (2,4,5)		
		Subgroup 1	Subgroup 2	Subgroup 3	Subgroup 1	Subgroup 2	Subgroup 3
MFGM-fglasso	Accu	1.000	0.9606	0.8481	0.9668	0.8457	0.9375
	TPR	1.000	0.7552	0.3545	0.8248	0.3428	0.4497
	FPR	0.000	0.0046	0.0003	0.0091	0.0001	0.0000
MFGM-ADMM	Accu	0.9937	0.9164	0.8181	0.9117	0.8201	0.9301
	TPR	0.8760	0.4621	0.2332	0.4203	0.2396	0.3645
	FPR	0.0001	0.0065	0.0022	0.0049	0.0016	0.0000
mixggm	Accu	0.9692	0.9490	0.8554	0.9343	0.8630	0.9016
	TPR	1.0000	0.7593	0.8557	0.7310	0.8102	0.5564
	FPR	0.0324	0.0188	0.1447	0.0312	0.1208	0.0557

### 3.2 Application to EEG data

Alcoholism is a common neurological disorder caused by the mutual effect of genetic and environmental factors. It not only damages the brain system but also leads to cognitive and mobility impairments [34]. It is of great importance to not only find a way that is reliable to distinguish alcoholics from normal subjects, but also recover the distinction of the brain patterns between alcoholics and normal subjects, which helps to explore the underlying mechanisms for alcoholism. Electroencephalogram (EEG) is a very effective tool for studying the complex dynamics of brain activities. It can visualize complex brain activities as dynamic outputs [35]. Therefore, it can be used to distinguish alcoholics from normal subjects based on the differences in the signals. A functional brain network accounts for the neuro-dynamical interactions between neural regions. Functional connectivity defines statistical interdependence between the dynamics of all pairs of the network nodes without taking into account causal effects [36]. Therefore, the analysis of the functional EEG data by mixture of functional graphical models is expected to depict the distinct brain networks in the two subgroups.

**Fig 2:**
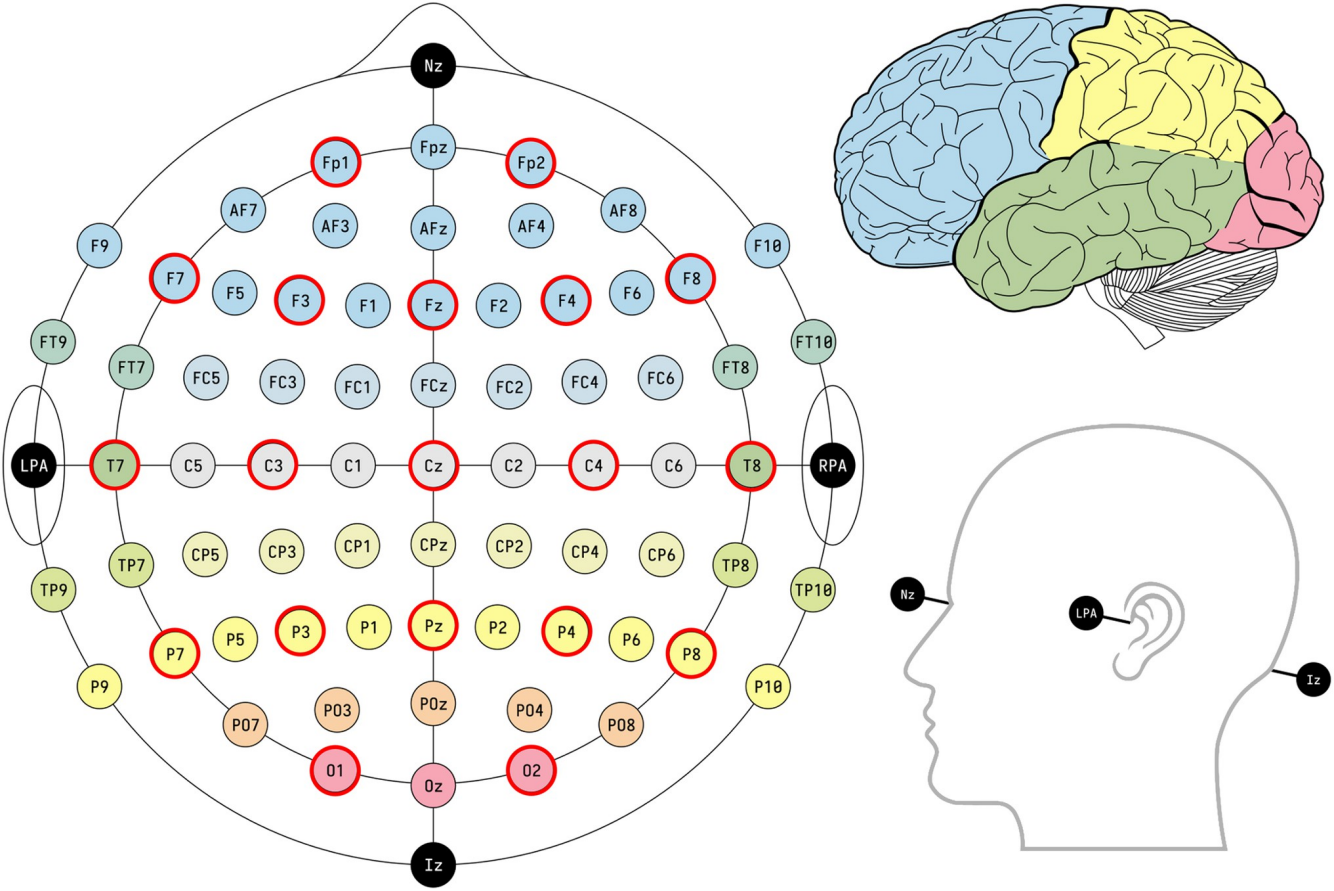
EEG channel system. The 19-channel montage specified according to the 10–20 International system (indicated by red circles). Picture by Laurens R. Krol.

We apply the proposed MFGM-fglasso algorithm along with MFGM-ADMM and mixggm algorithms on the EEG dataset acquired from the online UCI Knowledge Discovery in Databases Archive (https://kdd.ics.uci.edu/databases/eeg/eeg.html). Zhang et al. [37] describe in detail the data collection process. This data arose from a large study to examine EEG correlates of genetic predisposition to alcoholism. The study consisted of 122 subjects, of which 77 belonged to the alcoholism group and 45 to the control group. The data were initially obtained from 64 electrodes placed on the subjects’ scalps that captured EEG signals at 256 Hz during a one-second period. Each subject completed 120 trials under either a single stimulus (a single picture) or two stimuli (a pair of pictures) shown on a computer monitor. As the 64 electrodes were located at standard positions, to reduce the dimension of the data, we select the electrodes that detect signals in the 19-channel montage as specified according to the 10–20 International system (Fp1, Fp2, F7, F3, Fz, F4, F8, T7, C3, Cz, C4, T8, P7, P3, Pz, P4, P8, O1, O2) [38], which are depicted in Fig 2 by the red circles. Furthermore, referring to the cases considered in [17, 38], we focus on the EEG signals filtered at *α* frequency bands between 8 and 12.5 Hz that are acquired by applying the eegfilter function (R package eegkit) on the raw data. To remove the potential dependence between the measurements and the influence of different stimulus types, we only select observations under single stimulus for the use in this study [17, 20, 39]. Moreover, it shows that many studies used multiple samples per subject in order to obtain a sufficiently large sample, which violated the independence assumption inherent in most methods. Following the analysis in [17, 39], we average the valid band-filtered EEG signals across all trials for each subject.

First, the filtered EEG functional observations are fitted by using an *L*-dimensional cubic B-spline basis. The GCV method is used to choose the optimal dimension parameter *L*. Then the smoothed functions are each decomposed by *M*-truncated Karhunen-Loève expansion. Different from that in the simulation studies, the CV method always selects the highest value from the search grid as the harmonic number *M*, which leads to a very high dimension of the multivariate Karhunen-Loève expansion basis coefficient vector, making it too difficult for the following mixture model analysis. As the FPCA turns out that six principal components already explain more than 90% of the total variation in the signal trajectories for each node, we fix *M* = 6 as the truncation number for the Karhunen-Loève decomposition. The multivariate Karhunen-Loève expansion basis coefficient (principal component score) vectors ${\text{a}}_{i}^{M}$ with *M* = 6 are thus acquired for further mixture analysis assuming Gaussianity.

Similar to the simulation studies, we compared our MFGM-fglasso method with the MFGM-ADMM and mixggm algorithm. Again the Mclust function, acquired from the R package mclust, is applied to the multivariate principal component score vectors to obtain the initialization for EM algorithm. For the tuning parameter selection, values from 0.8 to 2.5 with an increment of 0.1 were tried. The optimal values were found to be λ_1_ = 2.2 and λ_2_ = 2.4.

In Table 3, clustering results of three algorithms are reported. Here, we can see that our proposed MFGM-fglasso method performed best in terms of finding two groups where Group 1 consists of most control subjects and Group 2 consists of most alcoholic subjects. Both MFGM-ADMM and mixggm found less distinctive groups compared to our proposed method.

**Table 3:** EEG clustering results for MFGM-fglasso, MFGM-ADMM, and mixggm.

**MFGM-fglasso**		Real Labels	
		Control	Alcoholic
Groups	Group 1	19	7
	Group 2	26	70
**MFGM-ADMM**		Real Labels	
		Control	Alcoholic
Groups	Group 1	25	15
	Group 2	20	62
**mixggm**		Real Labels	
		Control	Alcoholic
Groups	Group 1	20	24
	Group 2	25	53

**Fig 3:**
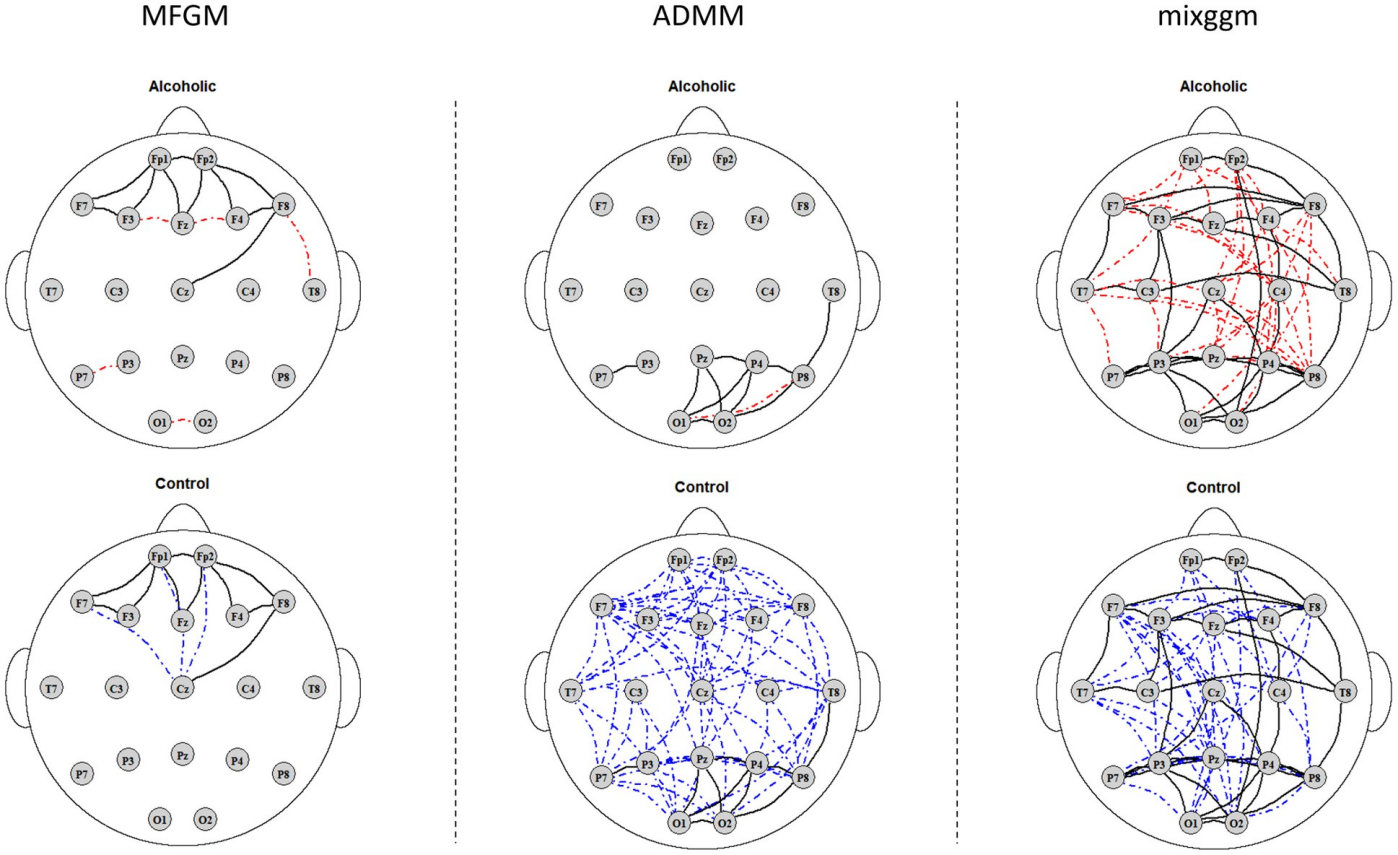
Comparison of conditional dependence structures in two subgroups. One group can be described as alcoholic and the other group can be described as control. Solid edges in black color represent common in both alcoholic and control; dash-dotted edges in red color represent specific in alcoholic; dash-dotted edges in blue color represent specific in control.

Fig 3 depicts the estimated brain nodes connection structures in each clustered group by the three methods. Our MFGM-fglasso method reveals that, in both subgroups, the electrode locations from the frontal region are densely connected, and the electrode locations from other regions of the scalp tend to be only sparsely connected. This is consistent with the findings reported by a functional graphical models study that analyzed the same EEG dataset [17]. Notably, while Qiao et al. [17] applied a functional graphical model separately to each true group, our approach analyzes data from both groups, simultaneously uncovering brain connectivity patterns and identifying the heterogeneous subgroups within the data. We also notice that the nodes connection structure in the frontal region in the alcoholic subgroup has an asymmetric pattern compared to a symmetric pattern in the control which echoes the findings from [38]. In addition, the Fz electrode-located region has a little more connection with the adjacent regions in the alcoholic subgroup than that in the control, but the Cz electrode-located region has less connection with the adjacent regions in the alcoholic subgroup than that in the control. Moreover, very sparse connections in the lower left Temporal region and Occipital region are revealed in the alcoholic subgroup compared to none in the control. The MFGM-ADMM algorithm shows distinction between the two subgroups. Very dense regional connections are found all over the whole brain in the control. In contrast, very spare regional connections are shown in the alcoholic subgroup except for the occipital region and the lower temporal regions. These findings do not align with the previous findings in the EEG study and this may suggest that the assumption of partial separability in MFGM-ADMM algorithm may not be valid for the EEG data analysis. Finally, the mixggm algorithm estimates super dense regional connections in both of the two subgroups which again does not aligns with previous studies. This might be due to the following reason. Taking the average of observations across the time interval for each node, ignoring the inherent functional nature in the data, could be invalid in EEG data analysis.

To sum up, our MFGM-fglasso method outperforms the other two competing methods in the real-world EEG data analysis, in finding distinctive two groups where one group represents the control group and the other group represents the alcoholic group and in estimating the heterogenous brain connectivity patterns.

### 3.3 Discussion

The main strength of our method lies in integrating mixture models with functional graphical models, which allows us to simultaneously detect heterogeneous subgroups within a population and estimate graph structures based on global correlation patterns. The promising performance of our approach is demonstrated through carefully designed simulation studies and its application to an EEG dataset studying alcoholism.

The simulation results also reveal that ignoring the functional structure of the data leads to suboptimal performance, and imposing the partial separability assumption on the precision matrix is similarly ineffective.

Our model assumes that the functional variables jointly follow a p-dimensional multivariate Gaussian process. If this assumption does not hold, alternative methods, such as copula Gaussian graphical models or nonparametric approaches, may be considered. Additionally, while we assume the number of clusters is known a priori, this is not always the case in practice. If the true number of clusters is unknown, model selection criteria such as BIC or the Integrated Classification Likelihood (ICL) can be used. However, due to the complex functional structure of graphical models, it remains unclear how to accurately compute the effective degrees of freedom for BIC [17].

Our method is also well-suited for estimating heterogeneous dependencies in human brain functional magnetic resonance imaging (fMRI) data and identifying subpopulations with shared brain connectivity patterns. For example, it can be applied to the ADHD-200 Global Competition dataset [40], which contains 776 resting-state fMRI scans from eight independent imaging sites. This dataset includes 491 scans from typically developing individuals and 285 from children and adolescents with Attention Deficit Hyperactivity Disorder (ADHD).

Moreover, our method is applicable to functional genomics, particularly in the analysis of gene expression data during disease progression, where patients may come from diverse backgrounds. Gene expression data are often represented as functional curves, with each gene’s expression measured at multiple time points. Our approach can uncover heterogeneous dependencies among genes within different patient subgroups, allowing for the identification of distinct gene interaction networks that evolve as the disease progresses.

## 4 Conclusion

We introduced the MFGM method, which combines mixture graphical models with functional data analysis (FDA) to generalize mixture graphical models from a vector-based to a functional context. Our MFGM method leverages an efficient EM algorithm that solves the log-likelihood maximization problem with a penalty, enabling the estimation of graphical model parameters for each subgroup. Additionally, we incorporate the fglasso algorithm within the EM framework to estimate the precision matrix. We believe that our approach, which not only clusters functional observations into subgroups but also uncovers heterogeneous conditional dependencies within each subgroup, significantly advances the methodology of high-dimensional graphical models.

The proposed method has the potential to expand the applicability of graphical models to a variety of complex data types, such as functional genomics, brain imaging, and longitudinal health data. By enabling more accurate modeling of heterogeneous dependencies, our method offers valuable insights into the underlying structures of high-dimensional data that are often missed by traditional methods.

Looking ahead, there are several promising avenues for future research. For example, extending our method to non-Gaussian settings could broaden its applicability, while further advancements in the selection of the optimal number of clusters could enhance model accuracy. Additionally, integrating our approach with other advanced machine learning techniques could improve its performance and scalability in real-world applications.

Ultimately, our method provides a novel strategy for analyzing complex functional data, offering new possibilities for understanding the intricate dependencies within high-dimensional datasets in various scientific and clinical fields.
